# Optimizing chromium removal from synthetic wastewater via electrocoagulation process with a natural coagulant (blended of eggshell powder and lime) using response surface methodology

**DOI:** 10.1016/j.heliyon.2024.e39234

**Published:** 2024-10-18

**Authors:** Firomsa Bidiraa, Endrias Adane Bekele, Werkne Sorsa Muleta

**Affiliations:** a Faculty of Civil and Environmental Engineering, Jimma Institute of Technology, Jimma University, P.O. Box-378, Jimma, Ethiopia; b Faculty of Materials Science and Engineering, Jimma Institute of Technology, Jimma University, P.O. Box-378, Jimma, Ethiopia; c School of Chemical Engineering, Jimma Institute of Technology, Jimma University, P.O. Box-378, Jimma, Ethiopia

**Keywords:** Electrocoagulation, Natural coagulant, Wastewater, Response surface methodology, Central composite design

## Abstract

The presence of chromium (Cr) in synthetic wastewater has become a serious environmental issue. Therefore, main aim of this work was to investigate Cr removal from synthetic wastewater via electrocoagulation (EC) with a natural coagulant using aluminum electrodes. The central composite design (CCD) of the response surface methodology (RSM) method was used to optimized the operating variables of solution pH (5–9), initial Cr concentration (225–475 mgL^-1^), reaction time (30–40 min), and applied current (0.35–0.55 A). The ANOVA results clearly shows that the quadratic model (p < 0.0001) was sufficient to the best predicting of the removal performance of Cr (R^2^ = 0.9994 for electrode distance of 0.5 cm and 0.9924 for 1 cm). The maximum removal (99.836 % for electrode distance of 0.5 cm, and 98.175 % for 1 cm) of Cr was achieved with optimized conditions of solution pH 7.053, initial Cr concentration 337.795 mgL^−1^, reaction time 37.148 min, and applied current of 0.505 A. From this finding, it was proved that the EC process assisted with natural coagulant is an efficient, and cost-effective method for the removal of Cr from synthetic wastewater.

## 1 Introduction

**Fig. 1:**
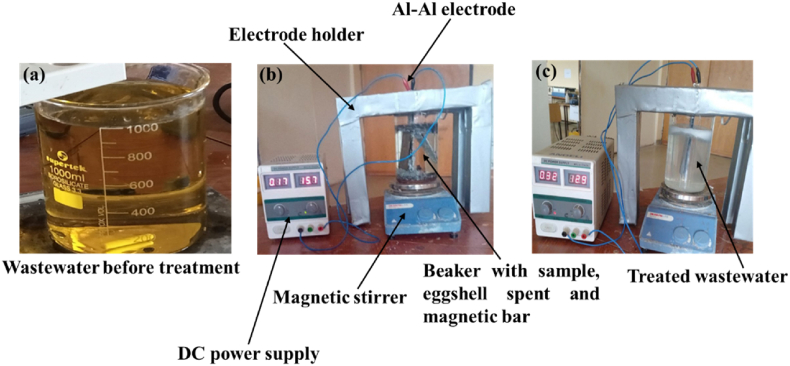
The experimental EC setup of the treatment method.

Today, water contamination is a significant issue affecting human's lives and nations' economic growth [1,2]. Water sustains life, influences human lifestyles, technology, language, and culture, but can be easily polluted through agricultural, industrial, and urban activities [3,4]. Everyone concern for water's value and its protection is paramount to avoid environmental pollution, making it human's responsibility to protect and conserve water for life [5,6]. Heavy metals, including copper, zinc, nickel, and chromium, are common pollutants in industrial wastewaters like metal plating and mining, posing high toxicity to aquatic environments [7,8]. Heavy metals are metallic elements in the periodic table, grouped 3–16 in periods 4 and higher, with atomic weights between 63.5 and 200.6 g mol^−1^ [9]. Metals have many advantages in daily life. Their contributions to the development of industries, modernization, and civilization. They might be essential as microelements to support the growth of plants or for maintaining the body's metabolism functioning properly for humans or animals. Metals can, however, cause environmental contamination and have detrimental impacts on living things if their concentrations rise above a certain point-even in small amounts [[10], [11], [12]]. Chromium (Cr) has different oxidation states, each with unique characteristics, such as trivalent (Cr (III)) being essential for carbohydrate metabolism, and hexavalent (Cr (VI)) being poisonous [13]. Cr (VI) is a highly toxic, mutagenic, carcinogenic, and teratogenic contaminant with strong oxidizing, toxic, and non-biodegradable properties. As per the World Health Organization's (WHO) rules, the highest permitted level of Cr (VI) in industrial wastewater discharged into surface water is 0.1 mgL^−1^, whereas the highest permitted level in drinking water is 0.05 mgL^−1^ [14]. For this reason, it is crucial to reduce Cr (VI) concentrations in industrial wastewater up to the acceptable limits before being discharged to the environment [15]. Cr (VI) can be removed from wastewater using a variety of treatment techniques, including chemical precipitation, co-precipitation, electrodeposition, membrane treatment, cementation, electrodialysis, biological reduction, electrocoagulation, ion-exchange, photocatalysis, adsorption, and biosorption. However, many technologies have limitations to various extents in practical applications due to the influence of high energy requirements, complex procedures, or the development of harmful by-products [7]. Among these, electrocoagulation (EC) is a green, cost-effective, low sludge generation and environmentally friendly method for treating wastewater, offering a simple and environmentally friendly solution without the need for additional chemicals [16]. EC processes involve oxidation, coagulation, and precipitation simultaneously using metal cations as coagulants in an electrochemical reaction. Iron (Fe) or aluminum (Al) electrodes are commonly used as anode. EC performance is influenced by water electrolysis, redox reactions of contaminants at the electrode surface, electrostatic effects, and ion migration. However, the main EC steps include, generation of cationic metal ions at the anode and hydroxide ions (OH^−^) at the cathode solution interface, transport of metal ions and OH- to the bulk solution, generation of metal hydroxide coagulants, and aggregation (agglomeration) of coagulants along with the contaminants [7,13,[17], [18], [19]]. The proposed mechanism of chemical reactions in the EC process is illustrated through the main reactions at the aluminum electrodes (1–5) [[20], [21], [22], [23], [24]]: (see Fig. 1)

Cathode reaction:

(1) $${2H}_{2}O+{2e}^{-}\to {2\text{OH}}^{-}+H_{2}\;\left(E^{0}\;\left(2H^{+}/H_{2}O\right)=-0.83\;V\right)$$

(2) $${2H}_{2}O+O_{2}+{4e}^{-}\to {4\text{OH}}^{-}\;\left(E^{0}\;\left(O_{2}/{\text{OH}}^{-}\right)=0.40\;V\right)$$

Anode reaction:

(3) $$\text{Al}\to {\text{Al}}^{3+}+{3e}^{-}\left(E^{0}\;\left({\text{Al}}^{3+}/\text{Al}\right)=-1.66\;V\right)$$

In site reaction:

(4) $${\text{Al}}^{3+}+{3\text{OH}}^{-}\to \text{Al}\left({\text{OH}}_{3}\right)$$

(5) $${Cr}^{3+}+{3e}^{-}\to {Cr\left(OH\right)}_{3}$$

Water treatment processes should be evaluated based on their effectiveness, ease of implementation, environmental friendliness, cost-effectiveness, and the required skilled manpower [25]. EC offers advantages over chemical coagulation or chemical flocculation, such as no secondary pollution risk, gas bubbles for pollutant collection, and easy automation. It produces clear, colorless, and odorless water, larger, stable flocs, and less sludge volume. EC also effectively removes even the smallest colloidal particles. However, it requires frequent replacement of sacrificial anodes, cathode passivation, complexity, high operational costs, and high current for effective treatment. Furthermore, its widespread adoption is hindered by limited access to affordable electricity and the need for alternative solutions. Further research is needed to develop EC for large-scale wastewater treatment [[26], [27], [28], [29], [30]]. On the other hand, Chemical coagulation (CC) removes pollutants by adding coagulant agents to neutralize the net surface charge, promoting aggregation, floc formation, and sedimentation. The CC process is traditionally performed using aluminum and iron coagulants like aluminum sulfate (Al_2_(SO_4_)_3_ ·18H_2_O), aluminum chloride (AlCl_3_), sodium aluminate (NaAlO_2_), ferric sulfate (FeSO_4_·7H_2_O), ferrous sulfate (Fe_2_(SO_4_)_3_·9H_2_O), and ferric chloride (FeCl_3_·6H_2_O) [31]. The demand for better coagulation performance has led to the search for natural coagulants as an alternative to chemical-based alternatives. Natural coagulants offer advantages such as renewability, biodegradability, nontoxicity, and cost effectiveness. They can come from plants, seeds, marine crustaceans, shellfish biomasses, and microbial organisms. Natural coagulants differ from inorganic and synthetic organic coagulants due to their polymeric structures, allowing charge neutralization and bridging for floc formation and impurity removal [[32], [33], [34], [35], [36], [37]]. Natural coagulants, despite their benefits, are not widely accepted in water and wastewater treatment due to industry confidence and uncertainties over their consistency and efficiency [38]. Food waste, primarily produced by homes and the food sector, is predicted to rise by 44 % between 2005 and 2025. Waste chicken eggshells, a type of solid waste, are often dumped into landfills. However, efforts are being made to transform waste eggshells into value-added products like adsorbents, coagulants, biocompatible materials, fertilizers, and catalysts [[39], [40], [41], [42]]. To the best of our knowledge no works have been done for the removal of Cr from synthetic wastewater via natural coagulant (blended of eggshell powder and lime) assisted EC process with the aid of response surface methodology (RSM), hence the significance and the novelty of this work. The study aimed the potential of natural coagulant-assisted electrocoagulation as a cost-effective and environmentally friendly solution for treating synthetic wastewater, thereby reducing its environmental impact. This study also used RSM-central composite design (CCD) method for optimizing the process parameters.

## 2 Materials and methods

### 2.1 Chemical reagents

All the reagents used in the study potassium chromate (K_2_CrO_4_, 99 %), sulfuric acid (H_2_SO_4_, 98 %), sodium hydroxide (NaOH, 99.8 %), sodium chloride (NaCl, 99 %), and hydrochloric acid (HCl, 37 %) were of analytical grade purchased from Sigma Aldrich (India). Distilled water was used to all stock solutions and synthetic wastewater. The pH of the artificial synthetic wastewater was adjusted by 0.1 M H_2_SO_4_ and 0.1 M NaOH.

### 2.2 Materials and equipment

The equipment used in this experiment includes oven dry, measuring cylinder, beakers, magnetic stirrer, weight balance, pipette, crucible, sieve, desiccator, filter paper, pH meter, UV Spectrophotometer (PerkinElmer Lambda 25, USA), multimeter, Voltmeter, polyethylene bottle, pestle, mortar, and spoon.

### 2.3 Preparation of synthetic wastewater

K_2_CrO_4_ with molecular weight of 194.1896gmol^-1^ was used in this study as a synthetic wastewater sample. The stock solution was prepared by dissolving 2 g of K_2_CrO_4_ in 2000 mL distilled water, then the final concentration was 2000 mgL^−1^. The experimental solutions were obtained by diluting the stock solution in accurate proportions to obtain different initial concentrations.

### 2.4 Experimental setup

The electro coagulation process was conducted by using a batch Plexiglas cylindrical reactor, with 1000 mL of artificial wastewater sample taken in a 2000 mL borosilicate glass beaker and rectangular sheets of Al electrodes utilized as anode and cathode electrodes, respectively, with dimensions of 14 cm (length) x 5 cm (width) x 0.2 cm (thickness) and a total anodes surface area of 70 cm^2^ would be used in the process. The electrodes were the connected externally to a constant direct current (DC) power supply to provide regulated current to the reactor. The distance between electrodes was adjusted to 1 cm and 0.5 cm in all experiments. The wastewater conductivity was adjusted by adding NaCl, while its pH was measured using a pH meter and adjusted by adding 0.1 M NaOH and 0.1 M H_2_SO_4_ solutions. The wastewater was agitated using a magnetic stirrer during EC at a speed of 250 rpm. The electrodes were thoroughly cleaned before each experiment, including washing them with distilled water, diluted HCl, and drying them. Finally, the concentrations of heavy metals in diluted samples were analyzed by UV–vis spectrophotometer (PerkinElmer, Lambda 25, Massachusetts, USA). The tests were conducted in triplicate and the mean data values were reported.

The efficiency of removing Cr was determined using the following Eq. (6) [43]:

(6) $$\text{Cr}\;\text{removal}\;\text{efficiency}\;\left(\%\right)=\frac{C_{o}-C_{t}}{C_{o}}\times 100$$

where C_o_ and C_t_ were the initial and residual concentration (mg. L^−1^), respectively, of the dissolved Cr ions at time t.

### 2.5 Operating cost

The EC process requires calculating an operating cost, including material (electrodes and energy) and essential expenses like sludge dewatering and disposal, to ensure low operational costs. This study considers energy and electrode material costs as major cost items in calculating the operating cost of wastewater treatment.

The operational cost for Cr removal was determined through Eq. (7) [44]:

(7) $$\text{Operational}\;\text{cost}\;\left(\text{OC}\right)=C_{\text{energy}}+C_{\text{electrode}}$$

where $C_{energy}$ and $C_{electrode}$ are energy and electrode consumptions respectively.

The electrical energy and electrode consumption for Cr removal were calculated by Eqs. (8), (9) [45,46]:

(8) $$C_{energy}\left({kgWhm}^{-3}\right)=\frac{VIt}{v\times 1000}$$

(9) $$C_{electrode}\left({kgm}^{-3}\right)=\frac{ItM}{nFv}=\frac{ItM}{nFv\times 1000}$$

where V is applied voltage (volt), I is current in A, t is treatment time (h), v is treated wastewater volume (m^3^), F is Faraday's constant (96,485 Cmol^-1^), M is the molar mass of Al (27 gmol^-1^), and n is the number of electron transfer (n = 3). The actual electrode consumption was determined by estimating the difference between electrode masses before and after treatment for a given volume at a specific time.

### 2.6 Preparation of natural coagulant (eggshell powder)

Chicken eggshells were collected from the restaurants of Stars in Jimma city, Jimma, Ethiopia. They were pretreated by boiling and drying at 105 °C for 12 h. The eggshells were ground and characterized using a sieve shaker machine to create a fine powder containing CaCO_3_. The powder was then calcined in a muffle furnace at 900 °C with 10 °C/min heating rate for 2 h, cooling, and obtaining CaO. The powdered eggshell was stored at room temperature for future use.

### 2.7 Proximate analysis

#### 2.7.1 Determination of moisture content

The moisture (water) content was determined by oven drying method. A weight of 10 g of eggshell powder was added to the crucible and weighed. The sample was kept in the oven at 105 °C for 3 h following the ASTM D2867-91. After that, the sample was taken out and kept in the desiccators. The weights sample was recorded before and after drying and burnt the samples into the oven and furnace respectively. Then the weight was measured and the moisture content was calculated as follow (10) [47]:

(10) $$\%\text{Moisture}\;\text{content}\;\left(\text{MC}\right)=\frac{D}{C-E}\times 100$$

where D is mass of crucible plus sample, C is mass of crucible plus dried sample, and E is the weight of the crucible.

#### 2.7.2 Determination of ash content

Eggshell powder samples were kept in a closed furnace and burnt at 650 °C for 2 h. The weight of the sample was taken with electronic balance. The percentage weight of residue gives the ash contained in the sample and its determined using equation (11) [48]:

(11) $$\%\text{Ash}\;\text{content}=\frac{D-E}{C-E}\times 100$$

where E is the weight of the crucible, C is the weight of the crucible and sample before firing; and D is the weight of the crucible and sample after firing.

#### 2.7.3 Determination of volatile matter

The volatile matter was determined according to ISO 562/197. 6 g of the sample was added to the crucible and weighed. It was kept in the muffle furnace at a temperature of 650 °C for 1 h. Then it was taken out and kept in the desiccators for half an hour to cool down [30]. Percent of volatile components (VC) in the sample was calculated using formula (12) [49]:

(12) $$\%\text{Volatile}\;\text{content}\;\left(\text{VC}\right)\;\frac{C-D}{C-B}\times 100$$

where B is denoted weight of crucible, C is weight of crucible plus sample before drying, and D is the weight of crucible and sample after firing.

#### 2.7.4 Determination of fixed carbon

The fixed carbon of the sample was calculated by subtracting the addition of moisture content, ash content (%) and, volatile matter (%) from 100 as equation (13):

(13) %Fixed carbon = 100% - (%MC + %AC +%VM)

### 2.8 Statistical analysis and modelling using RSM

In this study, response surface methodology (RSM) of central composite design (CCD) was used in the statistical analysis and modeling, which were carried out using Design-Expert software version 13.0 [50,51]. RSM is a set of statistical and mathematical techniques used to analyze and evaluate the interactive effect of multiple variables from empirical models to experimental data [52]. The central composite design (CCD) is a standard RSM experiment design that generates a quadratic response surface, analyzes factor interaction, and determines optimal parameters with minimal experiments [53]. In this work, four independent variables such as pH (5–9), initial concentration (225–475 mgL^-1^), treatment time (30–40 min) and applied current of (0.35–0.55 A) were used to determine the optimum removal of Cr from wastewater. The significant process variables considered in this study were as demonstrated in Table 1.

**Table 1:** Experimental design for EC process by RSM-CCD.

Factor	Name	Units	Type	Minimum	Maximum	Coded Low	Coded High	Mean	Std. Dev.
A	pH		Numeric	3	11	−1 ↔ 5	+1 ↔ 9.	7	1.82
B	Contact time	min	Numeric	25	45	−1↔ 30	+1 ↔ 40	35	4.55
C	Concentration	mg/L	Numeric	100.	600	−1↔225	+1↔475	350	113.71
D	Current	A	Numeric	0.25	0.65	−1↔0.5	+1↔0.55	0.45	0.091

The total number of experiments was calculated mathematically using Eq. (14) [54]:

(14) $${N=2}^{n}+2n+n_{c}$$

where N denotes the total number of experiments required, n denotes the number of elements and n_c_ denotes the center points. A total of 30 experimental runs were conducted in this work, 2^4^ = 16 cube points, six replications at the center point, and eight axial points.

The generalized second order polynomial model used for predicting the relationship between the independent variables and the response variable (Y) was as shown in Eq. (15) [55,56]:

(15) $$Y=\beta_{o}+\sum_{i=1}^{k}\beta_{i}x_{i}+\sum \sum_{i<j}\beta_{ij}x_{i}x_{j}+\sum_{i=1}^{k}\beta_{ii}x_{i}^{2}+\epsilon$$

where, Y is the response variable, $\beta_{o}\text{,}$ $\beta_{i}\text{,}$ $\beta_{ii}\text{,}$ and $\beta_{ij}$ are offset term, linear coefficients, quadratic coefficients, interaction coefficients, respectively. $x_{i}$ and $x_{j}$ are the independent variables.

## 3 Results and discussions

### 3.1 Proximate analysis

**Fig. 2:**
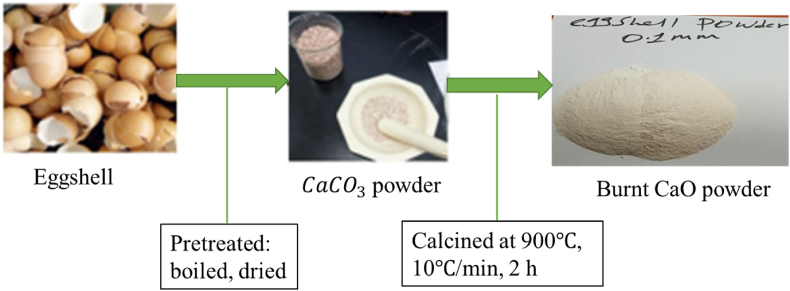
Synthesis of eggshell powder as a natural coagulant.

Table 2 shows the chemical characteristics of the eggshell powder (natural coagulant). From the results, it can be observed that the percent of moisture content, ash content, volatile matter, and fixed carbon were 5.23 %, 34.34 %, 26.98 %, and 41.24 %, respectively (see Fig. 2).

**Table 2:** Proximate analysis results of eggshell powder.

Chemical characteristics	(%)
Moisture	5.23
Ash	34.34
Volatile matter	26.98
Fixed carbon	41.24

### 3.2 Operational cost (OC)

#### 3.2.1 Effect of pH

**Fig. 3:**
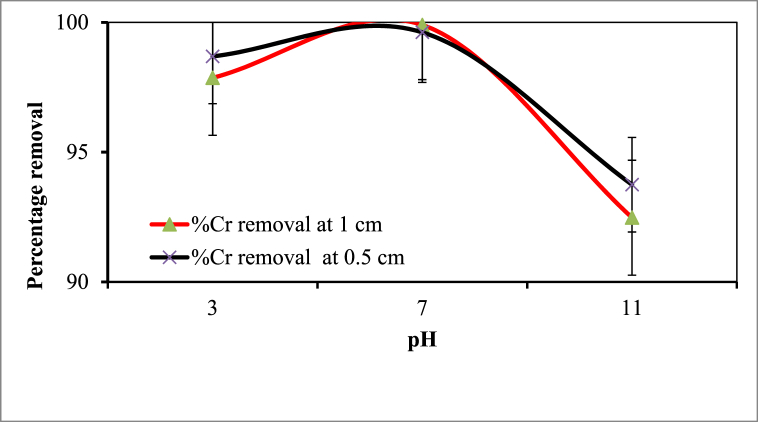
Effect of pH on the efficiency Cr removal (Experimental conditions: initial Cr concentration 350 mgL^−1^; reaction time 35 min; current density 0.45 A; coagulant dose of 0. 75 g).

The initial pH of a solution significantly impacts the efficiency of Cr removal in EC processes. Experiments were conducted with synthetic wastewater at pH ranging from 3 to 11 while others parameters kept constant (Cr concentration 350 mgL^−1^, reaction time 45 min, coagulant 0.75 gL^-1^ and applied current of 0.45 A). Fig. 3 showed that removal efficiency increased from 97.86 % to 99.89 %, 98.69 %–99.62 % with increasing pH up to 7, but decrease with increased the pH of the solution to 11 for both electrode distances (0.5 and 1 cm).

The highest Cr removal efficiencies were achieved at 99.89 % and 99.62 %, respectively, indicating the importance of pH in EC processes.

#### 3.2.2 Effect of initial Cr concentration

**Fig. 4:**
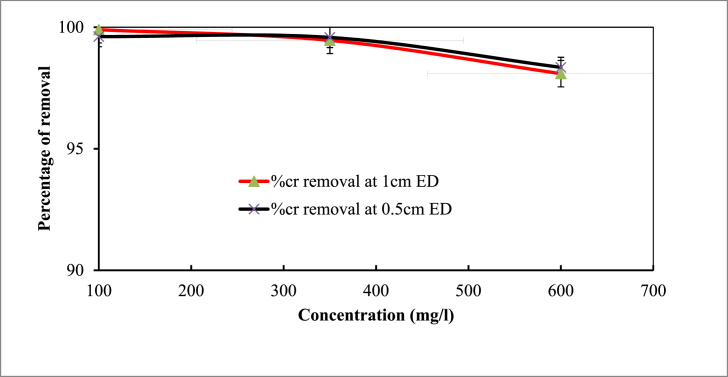
Effect of initial Cr concentration on removal efficiency (Experimental conditions: pH 7; reaction time 45 min; coagulant dose 0.75 gL^-1^; applied current 0.45 A).

Fig. 4 illustrates how the initial concentration of Cr affects the removal efficiency of the EC process from synthetic wastewater. It was clear that when concentration increases from 100 to 600 mgL^−1^ with constant parameters like pH 7, reaction time 35 min, applied current 0.45 A, and coagulant dose 0.75 gL^-1^, removal efficiency decreases from 99.62 % to 98.35 % for electrode distance of 0.5 cm and 99.90 %–98.09 % for 1 cm electrode distance, respectively. The maximum removal efficiency was achieved at 350 mgL^−1^.

#### 3.2.3 Effect of reaction time

**Fig. 5:**
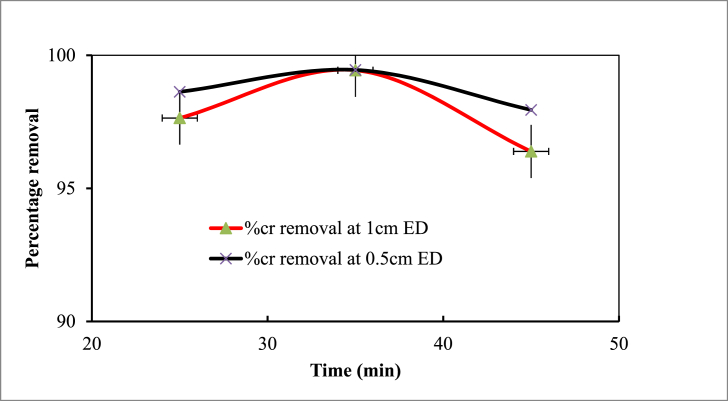
Effect of contact time on removal efficiency of Cr (Experimental conditions: pH 7; concentration 350 mgL^−1^; current 0.45 A; coagulant dose 0.75 gL^-1^).

The duration of electrocoagulation treatment significantly influences the effectiveness of Cr elimination, with a positive correlation between removal efficiency and time up to optimal duration. However, the removal efficiency decreased after this point due to the high abundance of flocs [57]. The experimental results show that reaction time significantly impacts the EC process for eliminating Cr at electrode distances of 0.5 and 1 cm were shown in Fig. 5. As reaction time increases, removal efficiency increases until 35 min, then decreases to 45 min, with all other factors constant. The maximum removal efficiency (99.45 % and 99.44 %) was achieved at a reaction time of 35 min.

#### 3.2.4 Effect of applied current

**Fig. 6:**
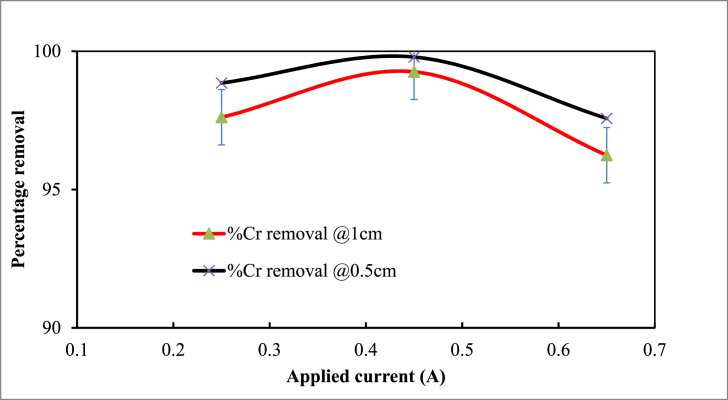
Effect of current on Cr removal efficiency (Experimental conditions: pH 7; concentration 350 mgL-1; reaction time 45 min; coagulant dose 0.75 gL-1).

Current is the electric current applied in amperes to an electrocoagulation cell or reactor, is a crucial element in the electrocoagulation process. Electrochemical reactions are driven by the applied electric current, which is related to hydrogen development at the cathode and oxygen discharge at the anode [58]. At a constant pH of 7, a 45-min reaction time, an initial Cr content of 350 mgL^−1^, and a coagulant dose of 0.75 gL^-1^, the impact of applied current was examined in the range of 0.25–0.65 A. The impact of applied current on the removal of Cr from synthetic wastewater is depicted in Fig. 6. As expected, it was found that with a current increase of up to 0.45 min, the removal efficiency increased from 98.85 % to 99.79 % (0.5 cm) and 97.62 %–99.26 % (1 cm).

### 3.3 RSM model and statistical analysis

#### 3.3.1 Analysis of variance and model fitting of RSM-CCD

The analysis of variance (ANOVA) was used to analyze the interaction between the proses variables and the response. The model F-value of 1724.54 for electrode distance (ED) 0.5 cm, and 139.81 for ED 1 cm, imply the model was significant. There was only a 0.01 % chance that an F-value this large could occur due to noise. Model terms with P-values less than 0.0500 indicate model terms were significant. A, B, C, D, AB, AD, BC, BD, CD, A^2^, B^2^, C^2^, D^2^ were significant model terms in this work. Values greater than 0.1000 indicate the model terms are not significant. The Lack of Fit F-value of 0.90 for 0.5 cm, and 3.79 for 1 cm, implies the Lack of Fit was not significant relative to the pure error. There was a 58.95 % (0.5 cm), and 7.72 % (1 cm) chance that a Lack of Fit F-value this large could occur due to noise [59]. ANOVA for the response based on independent variables for both ED (0.5 and 1 cm) was given in Table 3, Table 4.

**Table 3:** ANOVA of response surface quadratic model for Cr removal at ED = 0.5 cm.

Source	Sum of Squares	df	Mean Square	F-value	p-value	
A-pH	79.55	14	5.68	1724.54	<0.0001	Significant
B-Concentration	20.60	1	20.60	6251.82	<0.0001	
C-Reaction time	0.9955	1	0.9955	302.16	<0.0001	
D-Applied current	0.3760	1	0.3760	114.12	<0.0001	
AB	0.5227	1	0.5227	158.66	<0.0001	
AC	0.2965	1	0.2965	89.99	<0.0001	
AD	0.0044	1	0.0044	1.34	0.2648	
BC	0.1669	1	0.1669	50.65	<0.0001	
BD	0.7344	1	0.7344	222.92	<0.0001	
CD	0.4706	1	0.4706	142.83	<0.0001	
A^2^	0.1689	1	0.1689	51.27	<0.0001	
B^2^	54.35	1	54.35	16495.77	<0.0001	
C^2^	0.2279	1	0.2279	69.16	<0.0001	
D^2^	2.51	1	2.51	762.53	<0.0001	
Residual	0.4548	1	0.4548	138.05	<0.0001	
Lack of Fit	0.0494	15	0.0033			
Pure Error	0.0317	10	0.0032	0.8954	0.5895	not significant
Cor Total	0.0177	5	0.0035			
A-pH	79.60	29

**Table 4:** ANOVA of response surface quadratic model for Cr removal at ED = 1 cm.

Source	Sum of Squares	df	Mean Square	F-value	p-value	
Model	86.34	14	6.17	139.81	<0.0001	Significant
A-pH	17.96	1	17.96	407.19	<0.0001	
B-Concentration	5.41	1	5.41	122.76	<0.0001	
C-Reaction time	0.7273	1	0.7273	16.49	0.0010	
D-Applied current	1.83	1	1.83	41.52	<0.0001	
AB	0.3289	1	0.3289	7.46	0.0155	
AC	0.1376	1	0.1376	3.12	0.0977	
AD	4.63	1	4.63	104.89	<0.0001	
BC	1.86	1	1.86	42.24	<0.0001	
BD	0.2550	1	0.2550	5.78	0.0296	
CD	0.1862	1	0.1862	4.22	0.0578	
A^2^	48.69	1	48.69	1103.83	<0.0001	
B^2^	0.2134	1	0.2134	4.84	0.0439	
C^2^	5.75	1	5.75	130.42	<0.0001	
D^2^	5.75	1	5.75	130.27	<0.0001	
Residual	0.6616	15	0.0441			
Lack of Fit	0.5845	10	0.0585	3.79	0.0772	not significant
Pure Error	0.0771	5	0.0154			
Cor Total	87.00	29				

The coefficient of determination (R^2^) was 0.9994 for 0.5 cm, and 0.9924 for 1 cm closer to 1 indicating that the model was stronger and better predicted the response as shown in Table 5, Table 6. The adjusted R^2^ value was 0.9988 for 0.5 cm, for 0.98531 cm and the predicted R^2^ value was 0.9974 for 0.5 cm, and 0.9600. These R^2^ values explained that the model was significant [60,61]. Moreover, a small coefficient of variation (CV = 0.0584 for ED of 0.5 cm, and 0.2171for 1 cm) indicated a good deal of reflects the high reliability of the performed experiments and the accuracy of predictions. When measuring response values at the design space with the average prediction error, adequate precision yields a ratio larger than 4. An appropriate signal that can be used to navigate the design space was shown by the study's adequate precision values of 185.430 and 51.557 for ED of 0.5 cm and 1 cm, respectively [62].

**Table 5:** Statistical parameters obtained from the analysis of variances (ANOVA) for the models for Cr removal percentage from synthetic wastewater at ED of 0.5 cm.

Std. Dev.	0.0574	R^2^	0.9994
Mean	98.21	Adjusted R^2^	0.9988
C.V. %	0.0584	Predicted R^2^	0.9974
		Adeq. Precision	185.4301

**Table 6:** Statistical parameters obtained from the analysis of variances (ANOVA) for the models for Cr removal percentage from synthetic wastewater at ED of 1 cm.

Std. Dev.	0.2100	R^2^	0.9924
Mean	96.76	Adjusted R^2^	0.9853
C.V. %	0.2171	Predicted R^2^	0.9600
		Adeq. Precision	51.5574

The response functions with the determined coefficients explained by independent variables are presented in Eqs. (16) and (17).

Electrodes distance at 0.5 cm:

Y (%) = 99.76–0.9264A – 0.2037B + 0.1252C + 0.1476D - 0.1361AB

−0.1021AD – 0.2143BC - 0.1715BD

+ 0.1027CD – 1.41A^2^ – 0.0911B^2^ - 0.3026C^2^

−0.1288D^2^

Electrodes distance at 1 cm:

Y (%) = 98.63- 0.8651- 0.4750B - 0.1741C - 0.2762D + 0.1434AB - 0.5377AD

−0.3413BC + 0.1263BD - 0.1079CD - 1.33A^2^ - 0.0882B^2^

−0.4580C^2^ - 0.4577D^2^

**Fig. 7:**
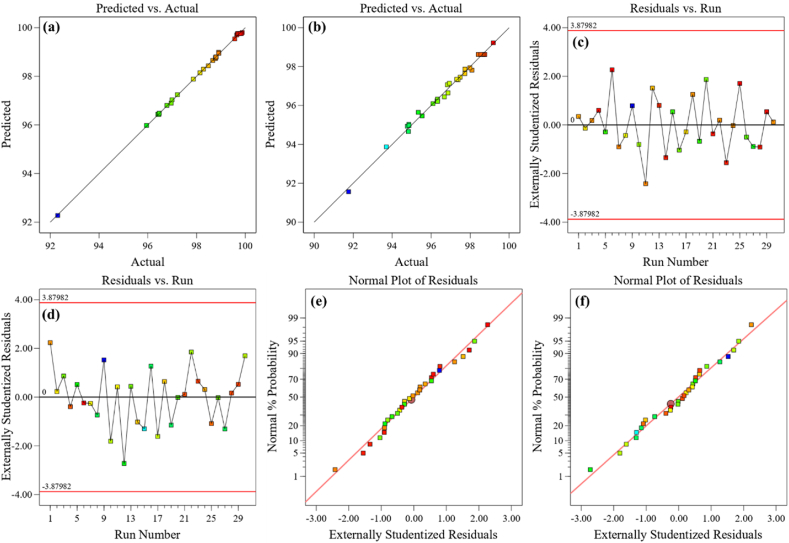
Model fittings plots for the removal of Cr from synthetic wastewater.

The study of residuals is an important technique for diagnosing and anticipating the behavior of the proposed model. The model fitting responses are shown in Fig. 7a–f. The graphs of actual vs predicted data acquired from the EC of Cr removal were depicted in Fig. 7a for ED 0.5 cm, and b for ED 1 cm, respectively. From this plot, we can say that experimental removal data and predicted values are nearly identical and the distribution of data trend was found to be excellent. The plots of residuals vs run were illustrated in Fig. 7c and d. The residuals in the plots were fluctuated in a random fashion around the centerline and within the range. As a result, the suggested model is satisfactory and accurately represents the EC process. Fig. 7e and f depicts the results of the normal values and the actual statistic of the suggested model for Cr removal. It is seen that Cr removal exhibited relatively well-behaved residual and normal distributed because it followed the straight line.

#### 3.3.2 Interaction study of process variables

**Fig. 8:**
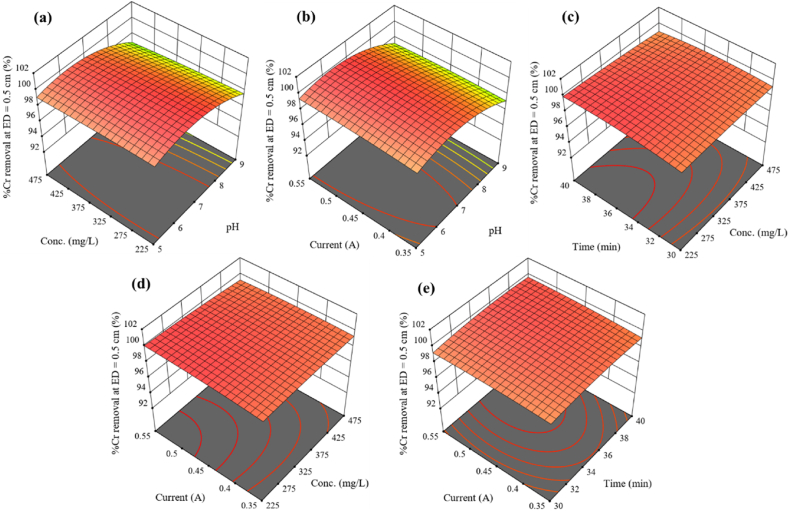
Three-dimensional (3D) plots for Cr removal: effects of a) Concentration and pH, b) current and pH, c) time and concentration, d) current and concentration and e) concentration and contact time.

Three-dimensional (3D) surface plots help to investigate the effect of the independent variables and their interactions on the response and were illustrated in Fig. 8a–e. The combined impact of the initial Cr concentration and solution pH on Cr removal uptake is shown in Fig. 8a for a reaction period of 35 min and an applied current of 0.45 A. The removal efficiency decreased from 99.32 to 97.73 % when concentration (225–475 mgL^−1^) and solution pH (5–8) increased. The removal of Cr in the EC process was significantly influenced by the interaction between solution pH and applied current. Fig. 8b showed a positive effect was observed when the applied current was increased from 0.35 to 0.55 A and the pH was increased from 5 to 7. The percentage removal of Cr increased from 98.93 to 99.51 % with an increase in current and pH up to 7, but diminished as the solution pH increased.

The interaction effect of Cr concentration and reaction time on percentage removal at constant solution pH (7) and applied current (0.45 A) was presented in the form of 3D surface plots as shown in Fig. 8c. It was revealed that the removal efficiency of Cr increased from 99.24 to 99.96 % with an increase in reaction time from 30 to 40 min. On the other hand, the increase in Cr concentration decreased in percentage removal. Fig. 8d demonstrates the interaction effect of the applied current and initial Cr concentration on the removal percentage of Cr from synthetic wastewater in an EC process. From the plot, it was observed that the removal efficiency increased with an increase in applied current from 0.35 to 0.55 A. On the contrary, with the increase in the initial Cr concentration, the percentage removal decreased. Fig. 8e shows the response surface plot of Cr removal efficiency as a function of applied current and reaction time. As the applied current increases from 0.35 to 0.55 A, the percentage removal also increases from 99.18 to 99.35 %. The interaction of applied current and reaction time significantly affects Cr removal, with a p-value of 0.0001.

#### 3.3.3 Optimization of process parameters

**Fig. 9:**
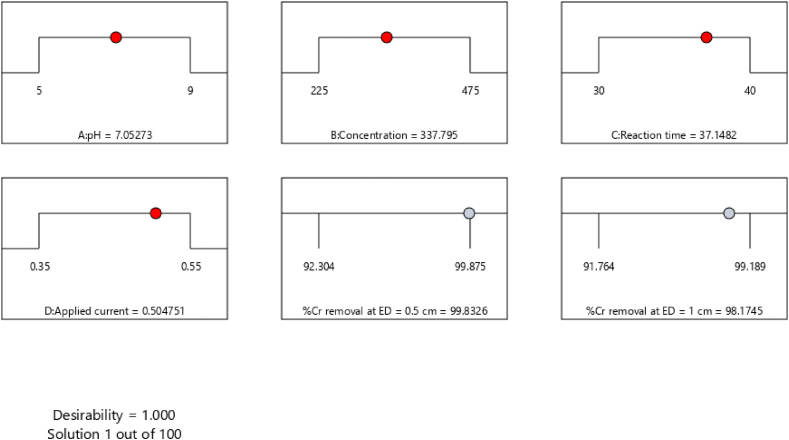
The optimization graph displays the best-predicted values for the maximum removal of Cr (%) and desirability function.

Using CCD-RSM approaches, the highest removal efficiency was prioritized as a desirable aim while optimizing the operation variables. The optimal parameters included a solution pH of 7.053, initial Cr concentration of 337.795 mgL^−1^, reaction time of 37.148 min, and applied current of 0.505 A and optimization results were shown in Table 7 and Fig. 9. The maximum removal efficiency was achieved at 99.833 % for 0.5 cm ED and 98.174 % for 1 cm ED, accurately describing the experimental model and intended conditions with a desirable value of 1.00 (see Table 8).

**Table 7:** Optimal conditions of the selected factors for Cr removal at ED = 0.5 cm and 1 cm.

Optimized process parameters				Removal efficiency (Y, %)		
pH	Conc.	Time	Current	0.5 cm	1 cm	Desirability
7.053	337.795	37.148	0.505	99.833	98.174	1

**Table 8:** Comparison of Chromium ion removal from aqueous solutions by other researcher with the current work.

S.No.	Electrode material	Wastewater type	Current density, Current or Voltage	Removal Efficiency	Reference
1	Fe and Al	Aqueous solutions	200 A/m^2^	100	[63]
2	Fe	Aqueous solutions	1 A	56.3	[64]
3	Fe	Aqueous solutions	10.02 A/m^2^	94.97	[65]
4	Al	Aqueous solutions	9.14 V	91	[66]
5	Cu	Aqueous solutions	41.32 A/m^2^	95.21	[67]
6	Al	Aqueous solutions	0.4667 A/m^2^	99.46 & 99.62	This work

### 3.4 Strengths and limitations of the research

The study makes an important contribution to wastewater treatment. Combining electrocoagulation with a natural coagulant made up of powdered hen eggshell provides an economical and sustainable alternative. The detailed experimental design of the study, which includes 30 separate runs, assists in determining the ideal conditions for chromium removal. The treatment method is made more sustainable by using hen eggshell powder, a naturally occurring and easily accessible substance that also minimizes costs and has less of an adverse environmental effect. The comprehensive examination of the study guarantees an accurate comprehension of the relationships between various variables, resulting in a removal procedure that is more effective and efficient. The study's novel electrocoagulation with natural coagulants combination, precise optimization process, and potential for real-world wastewater treatment applications are what make it practically relevant.

The research on the removal of chromium from synthetic wastewater by electrocoagulation in combination with a natural coagulant (lime and powdered hen eggshell) has several drawbacks. The complex nature of actual industrial or municipal wastewater, which can contain large concentrations of sludge and suspended particulates, may not be entirely represented by synthetic wastewater. Furthermore, there are problems with scaling for natural coagulants, and the quality of preparation and source can affect how effective they are. Depending on the particular types of chromium present and their elimination, the study's efficiency may vary. Additionally, the optimization process requires measurement and accurate adjustment for operating variables as well as variable interactions. the technology was applicable where there was electric power and skilled manpower for the operation and preparation of adsorbent.

## 4 Conclusion

The primary driving force behind the development of innovative effluent treatment methods is environmental concerns. In the present work, the efficiency of EC was investigated for the removal of Cr from synthetic wastewater with a natural coagulant using Al electrodes. The results obtained were extremely encouraging and have shown that EC is an appropriate method of treatment for synthetic wastewaters. The RSM based on CCD method was applied to find the optimal process variables on the removal efficiency. Analysis of variance (ANOVA) results for the second-order polynomial model derived from the EC process indicated that the model was able to predict the response with a good correlation between the empirical and predicted data. By adopting the design of experiments, statistically, it was optimized that the maximum removal (99.836 % for ED of 0.5 cm, and 98.175 % for 1 cm) of Cr can be achieved via pH 7.053, initial Cr concentration 337.795 mgL-1, reaction time 37.148 min, and applied current of 0.505 A. EC proposes advanced technologies for water and wastewater treatment, but the process is not fully optimized and more research is needed for global implementation. Strengths of research include investigating process costs, presenting mechanisms, and determining the importance of variables affecting removal efficiency. This study suggests that EC integration with natural coagulant for toxic Cr removal, a cost-effective wastewater treatment framework and minimizing negative impacts on water quality by removing harmful metals.

## CRediT authorship contribution statement

**Firomsa Bidiraa:** Software, Resources, Formal analysis, Data curation. **Endrias Adane Bekele:** Validation, Software, Formal analysis, Data curation, Conceptualization. **Werkne Sorsa Muleta:** Writing – review & editing, Writing – original draft, Visualization, Supervision, Conceptualization.

## Data availability statement

All data are available within the manuscript.

## Ethics approval

We declare that this manuscript is original, has not been published before, and is not currently being considered for publication elsewhere.

## Funding

This work was financially supported by 10.13039/501100005068Jimma University, Jimma Institute of Technology.

## Declaration of competing interest

The authors declare that they have no known competing financial interests or personal relationships that could have appeared to influence the work reported in this paper.
